# Effects of whale-based tourism in Vava’u, Kingdom of Tonga: Behavioural responses of humpback whales to vessel and swimming tourism activities

**DOI:** 10.1371/journal.pone.0219364

**Published:** 2019-07-05

**Authors:** Lorenzo Fiori, Emmanuelle Martinez, Mark B. Orams, Barbara Bollard

**Affiliations:** 1 Institute for Applied Ecology New Zealand, School of Sciences, Auckland University of Technology, Auckland, New Zealand; 2 Applied and Environmental Sciences Department, NorthTec, Whangarei, New Zealand; 3 TriOceans, Marine Research and Technology Institute, Bay of Islands, New Zealand; 4 School of Sport and Recreation, Auckland University of Technology, Northcote, New Zealand; 5 Sustainability Research Centre, University of the Sunshine Coast, Queensland, Australia; University of Minnesota, UNITED STATES

## Abstract

Vava’u, Kingdom of Tonga, is a well-established whale-watching destination in the South Pacific. Between July and October, the waters around the archipelago represent one of the most important breeding grounds for Oceania humpback whales (*Megaptera novaeangliae*). The Tongan government allows tourist swimming activities with whales and tour operators strongly promote the practice of swimming-with-whales, focusing primarily on mother-calf pairs. However, there is increasing evidence, derived from empirical research on swim-with-cetacean tourism, that this kind of interaction affects cetacean behaviour and can lead to negative effects on the cetaceans involved. This study represents the first assessment of humpback whales’ behavioural responses to vessel and swimmer approaches in Vava’u. Fifty-six surveys took place during the 2016 and 2017 whale breeding seasons aboard dedicated research and tour vessels. Whale dive time, number of reorientation events, and respiration rates were documented in both the absence and presence of boats and swimmers. Vessel approach type, swimmer placement, and whale avoidance responses were also recorded. Results indicate that the average diving time and the proportion of time spent diving in the presence of swimming activities increased significantly for mother-calf pairs (*F*_*2*,*36*_ = 18.183, *P* < 0.001; *F*_*2*,*36*_ = 5.462, *P* = 0.009, respectively). Moreover, avoidance responses of whales towards tour vessels were observed for one third of vessel approaches (33.5%) and the avoidance rate was significantly affected by the boat approach type (95% CI: 20.7–69.2%, z = 3.50, *P* < 0.001). Finally, low levels of compliance to the existing Tongan swim-with-whales regulations were documented, in particular the stipulated whale resting time between interactions with tour operator vessels and swimmers was often not respected (38.4%). Vava’u is an important calving ground for the Oceania humpback whale population and these findings should be carefully considered by stakeholders in Tonga and at other locations where swim-with-whales opportunities are being undertaken. Effective strategies to reduce the risk of detrimental effects on the whales targeted by swimming activities, especially mother-calf pairs, are needed.

## Introduction

The whale-watching tourism industry’s focus on humpback whales has escalated worldwide in the last three decades according to O'Connor et al. [1]. This global trend has been supported by the recovery of some whale populations [2] and the increasing public demand for tours offering close interactions with whales [3,4]. Consequently, tourism activities focussing on humpback whales have increased, both in regions representing important breeding grounds for this species and on migration routes [5].

The Kingdom of Tonga promotes swimming activities with humpback whales during their breeding season, with mothers and calves being the primary target [6]. The growth of whale-based tourism in Tonga since the 1990s now represents a major source of foreign income for the nation [6,7]. Vava’u, a northern archipelago of the Kingdom, is where the first swim-with-whales commercial activity started in 1993 [8]. By 2017, the island group had 20 commercial tour operators offering in-water encounters with humpback whales (Tongan Ministry of Tourism, personal communication, October 8, 2017), more than any other whale-watching destinations worldwide [5]. Each operator can obtain up to two licences for swimming activities and, consequently, have two tour vessels operating at the same time. In addition, a low level of compliance with the Tongan existing regulations has been reported in the past [9].

Swimming activities with whales are still prohibited in most countries where whale-watching occurs [5] and the scientific community has urged the need for a more precautious approach to the management of commercial tourism operations [10–12]. There is widespread concern amongst the scientific community (e.g., [13–19]), that swim-with-cetaceans tourism can disrupt vital behaviours and cause avoidance responses in the targeted cetaceans. For example, increases in the number of dives (also referred as vertical avoidance) have been reported for humpback whales [20–24] and sperm whales (Physeter macrocephalus) [25] exposed to approaches from tourism vessels. Humpback whales [24,26,27] also show a less direct swim path (a behaviour known as horizontal avoidance) when approached by whale-watching boats. Similar responses have been observed during swim-with trials conducted with humpback whales in Western Australia [18]. Moreover, the need to place swimmers in close proximity to the whales can encourages types of vessel approaches that are less tolerated by the whales and have been judged to be highly invasive [18,28,29]. For instance, tour operators may increase their approach speed to overtake the whales and position the boat in the whale’s path of travel to increase the success of the swim-with activity for the tourists [19]. This behaviour has been defined as a “J approach” by Scarpaci et al. [30] and it is strongly discouraged by whale-watching regulations worldwide [31].

Some studies have also demonstrated that short-term responses to vessel approaches can lead to long-term effects for dolphins at both the individual and population levels [32–35]. However, only a few studies have focused on the behavioural responses of baleen whales to swim-with tourism activities [8,19,36]. Responses to swimmer approaches seem to depend on the targeted species. Significant changes in the behavioural budget have been observed in southern right whales (*Eubalaena australi*s) in Península Valdés, Argentina [19], while dwarf minke whales (*Balaenoptera acutostrata*) appear to deliberately approach vessels and swimmers in the Great Barrier Reef, Australia [36]. This research is important because there are a growing number of locations which are opening up swim-with-humpback whales tourism (eg., Queensland, Western Australia, Niue, French Polynesia, the Dominican Republic) [5,37,38]. Vava’u, Tonga is an appropriate location to conduct this research because it has a long-established and intensive swim-with-humpback whales tourism sector [6,7,9] and, importantly, these commercial swim-with-whales operations take place in a humpback whale breeding and calving ground [39]. In addition, the Tongan humpback whale sub-population still shows little signs of recovery after the cessation of whaling [40–42].

This study represents an assessment of the behavioural responses of humpback whales to vessel and swimmer approaches in Vava’u, Kingdom of Tonga. First, the effect of vessel and swimmer approach type chosen by tour operators on whales’ reaction were investigated. The hypothesis was that, if the approach type would not influence the whale response to the approach, the avoidance rate would not differ significantly between different type of approaches. Secondly, whales’ diving, respiration, and reorientation parameters were quantified and the effects of swimming tourism activities on these response variables were assessed. The hypothesis was that, if vessel and/or swimmer presence (experimental situations) do not affect whales’ behaviour, then we would not detect significant differences for dive time, diving rate, proportion of time spent diving, respiration rate, and reorientation rate in comparison with the absence of tourism activities (control situations). Finally, the level of compliance with regulations was evaluated.

## Methods

### Study site and species

**Fig 1:**
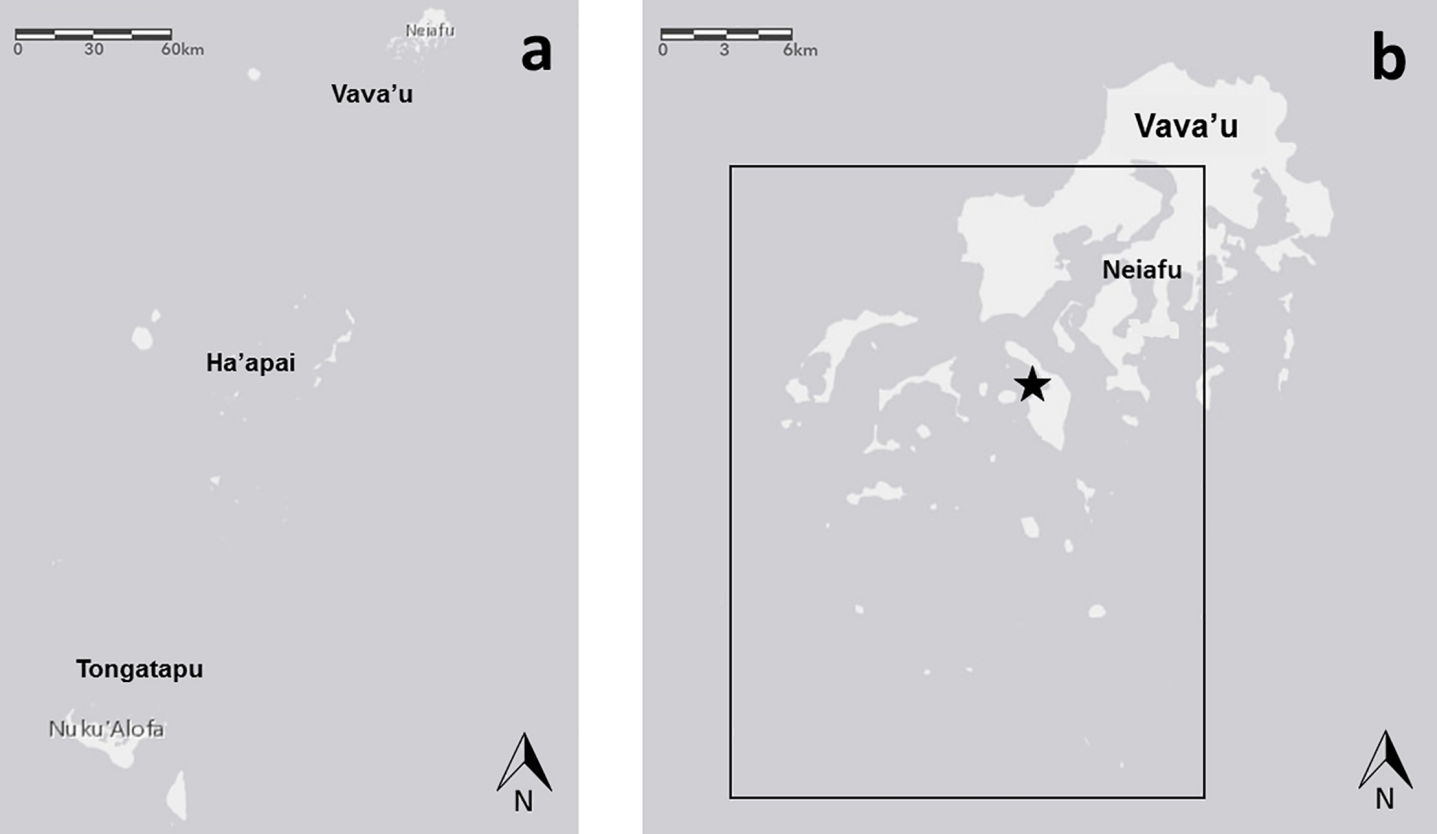
Vava’u, Kingdom of Tonga. (**a**) The Kingdom of Tonga is constituted of three main island groups and the remote Niuatoputapu islands. The study was conducted off Vava’u Island (18°39’S, 173°59’W), (**b**) on the southern side (rectangle). Neiafu (18°39’S, 173°58’W) is the main township and the harbour from where tour vessels departed. Port Maurelle (18°42’S, 173°01’W), the anchorage from where most of the research dedicated surveys departed, is also indicated (star).

The study was conducted during humpback whale breeding seasons (between July and October) in 2016 and 2017 on the South side of Vava’u (18°39’S, 173°59’W), Kingdom of Tonga (Fig 1A and 1B). This area has been documented as an important breeding ground for Oceania humpback whales [41]. That is, the waters between the islands of the archipelago represent the major calving site for the Tongan sub-population [39]. The study was undertaken under the permit MOT 4/3 issued by the Tongan Ministry of Tourism.

### Survey design

Control data (samples in the absence of tour vessels and swimmers) were collected in the absence of commercial tour vessels or private vessels within a 1,000 metres radius of the focal group of whales. Data were gathered aboard a research dedicated 11 metres sailing trimaran and a 6 metres powerboat (powered by a 2-stroke Mercury 25 horse power) travelling at speeds of less than 10 knots, depending on sea and wind conditions. The observer’s eyes were at a height of 2.5 meters above the surface of the water. The vessels were operated from an anchorage in Port Maurelle (Fig 1B). Surveys took place only in good weather conditions (Beaufort and Douglas Sea State < 4, no rain) and involved a skipper, the primary researcher and a trained observer. Once a group of humpback whales was sighted, the vessel approached and moved parallel to the heading of the group at idle speed (less than 5 knots) to minimise effects on the whales’ behaviour [23,43]. In accordance with Tongan regulations for non-whale watching vessels, the vessel was stopped at 300 metres from the whales. Distances were measured in metres with a Rangemaster 1600-B laser rangefinder (Leica Camera, Wien, Austria). Control observations lasted 30 minutes, then the research vessel left the area to search for other whale groups. This protocol was based on other studies on the behavioural responses of humpback whales [18] and southern right whales [19] to swimming activities. A 30 minutes observation was deemed appropriate to gather sufficient data about whale behaviour at the surface and the surface respiration pattern, also considering the maximum observed dive time of humpback whales in Vava’u during this study. Moreover, this protocol aimed to minimise potential disturbance to the whales, especially mother-calf pairs.

Experimental data (samples in the presence of tour vessels and swimmers) were collected by the primary researcher and the observer from two swim-with-humpback whale tour operator vessels, hereafter referred to as vessel A and vessel B. As for the research vessels, the observer’s eyes were at a height of 2.5 meters above the surface of the water. The two tour boats share similar sizes (10 metres in length) but they differ substantially in terms of engines and hydrodynamics. Vessel A is a catamaran design and it is gasoline-powered by two 4-stroke Yamaha 250 horse power outboard engines. Vessel B is a single hulled boat and is powered by two inboard diesel engines (Cummins 350 horse power). The boats were voluntarily offered by tour operators to be used as platform of opportunity for this research. Therefore, researchers had no control over the type or speed of vessel approach to whales, minimum distance to the whales and placement of swimmers into the water. As per control observations, a 30-minute protocol was followed to collect data during swim tourism activities.

### Focal group follows and data collection

An encounter between a tour vessel and whales was defined as beginning when a vessel was approximately 1,000 metres from the focal group of whales. A focal group was represented by one or more whales within 100 metres from each other, coordinating their behaviour and moving in the same direction [21,44,45]. Date and time, location (latitude and longitude using GPS), sea-state (Beaufort and Douglas scales), weather, wind speed and direction, and depth, were recorded at the beginning of each encounter. In addition, the initial composition of the whale group was assessed. A calf was identified as a whale of less than 50% body length of an adult (full size) whale in close proximity, which was defined as mother [21]. An adult whale consistently accompanying a mother and calf pair was defined as an escort [46].

The behavioural state of the focal group of whales was defined as the behaviour in which more than 50% of the whales were involved, as described for marine mammal focal group follow protocols by Mann [47]. Five mutually exclusive and cumulatively inclusive behavioural states [48] were defined to describe whale behaviour during the encounters: resting, travelling, surface-active, socializing and feeding [18,23,49] (Table 1). Feeding behaviour was unlikely to be observed due to the scarce distribution of humpback whale prey in tropical breeding grounds [50,51]. An H2A-XLR hydrophone (Aquarian Hydrophones, Anacortes, WA USA) was also deployed from both research and tour boats to detect potential singing behaviour, typically associated with prolonged dives. However, singing behaviour was not included in the ethogram used by the observer as the visual detection of this behavioural state is not possible.

**Table 1:** Definitions of behavioural states of individual humpback whales. Adapted from Sprogis et al. [18], Stamation et al. [23] and Di Clemente et al. [49].

**Resting (R)**	Whale is motionless and horizontal at the water surface, may be also drifting or slightly below the water surfacing only to breathe.
**Travelling (T)**	Whale is travelling from location to location with persistent, directional movement making noticeable headway along a specific compass bearing at a constant speed and may leave rows of “fluke-prints” at the surface.
**Surface-Active (SA)**	Whale is causing white water at the surface by rolling, breaching, spy hopping, caudal fin, pectoral flipper or head slapping.
**Socialising (S)**	Whale is actively rubbing, touching, chasing or circling around another whale. Underwater blows can be observed.
**Feeding (F)**	Whale is rapidly emerging with the ventral plates extended. The body at the act of surfacing can be lateral or vertical.

Whale responses to vessel and swimmer approaches were categorised as either “avoidance” or “no avoidance”. An avoidance response was defined as a movement away from the approaching vessel or swimmers [23]. A “no avoidance” response included any other potential category (i.e. attraction and neutral). The boat approach type was recorded as direct, parallel or J (Table 2) [30]. The distance between vessel and the closest whale was measured by the primary researcher using the laser rangefinder every time whales were present and visible at the surface. Similarly, whales joining or leaving the focal group, as well as the number and names of tour boats arriving or departing in the area (1,000 metres around the focal group), were recorded through the encounters.

**Table 2:** Definitions of vessel approach type [30].

**Parallel**	The tour vessel is positioned to the side of the focal whale group, parallel to the direction of travel.
**Direct**	The tour vessel is manoeuvred directly to the middle of the focal whale group. This may happen from any direction with respect to the heading of the whale group.
**J**	The tour vessel travels parallel to the focal whale group direction of travel, overtakes the whales and is then turned in front of the group.

Swimmer placement (Table 3) was generally associated with the tour vessel approach type: “in path” (during vessel J approaches), “line abreast” (during vessel parallel approaches) and “around boat” (during vessel direct approaches) [16].

**Table 3:** Definitions of swimmer placement type. Adapted from Constantine [16].

**Line abreast**	On the side, parallel to the direction of travel of the focal whale group, slightly ahead of the whales.
**In path**	In the path of travel of the focal whale group.
**Around boat**	The tour boat is stationary with the focal whale group circling around it.

Dive time (seconds), number of dives, respirations (number of “blow” exhalations during a surfacing period), and group reorientation events (change in swim direction of 90° or more in respect to the original heading direction) were recorded continuously [52]. Average dive time (total dive time × number of dives^-1^), diving rate (dives × hour^-1^), and respiration rate (blows × individuals^-1^ × minute^-1^) were calculated. As asynchronous diving behaviour was observed for mothers and calves, a focal individual (i.e., the mother) was selected to record the dive time. This decision was made as it was problematic for the observer to record the dive time of two or more whales while collecting other data at the same time. Therefore, in the case of groups containing a calf, the mother was chosen as the focal individual (it was always the case that the calf and the escort, if present, followed the adult female mother, and she was readily recognisable by the observer). Multiple mother-calf pairs were never observed in the same group of whales, neither they were accompanied by more than one other individual (i.e., escort).

### Statistical analysis

#### Avoidance responses to vessel and swimmer approaches

Generalized Linear Models (GLM) were used to test the hypothesis that the vessel approach type had no effect on whales’ response. That is, the presence or absence of an avoidance response was modelled as a function of approach type (AT), vessel A or B (V), distance between vessels and the closest whale (DI) and water depth (DP), using a binomial distribution with logit link function. Two two-way interactions were also tested (AT × V and V × D). Due to the low sample size of direct approaches, only parallel and J approaches were considered for analysis. GLMs were compared using Akaike’s Information Criterion (AIC). The best fitting model had the lowest AIC and models falling within two units were considered to have substantial support [53]. That is, AIC assist in the identification of the model that provides more information using less parameters. Models falling within the two-unit range were considered to provide an equal amount of information and parameters were further evaluated to choose the most parsimonious model. Any significant effect of a parameter on the avoidance response occurrence was further investigated by comparing the avoidance rate with a z-test for proportions. Confidence intervals (95%) were also calculated.

Similarly, GLMs with binomial distribution and logit link function were used to test the dependence of whales’ response to swimmer approaches. The presence or absence of avoidance response was modelled as a function of swimmer placement (SP), vessel (V), distance of the boat from the whales at swimmer drop (DD) and water depth (DP). Two two-way interactions were also considered, swimmer placement × vessel and vessel × distance of the boat from the whales at swimmer drop (SP × V and V × DD). Due to the low number of swims “around the boat”, only “line abreast” and “in path” placements were analysed. In addition, “line abreast” approaches were modelled to test the whale’s response as a function of vessel (V), vessel distance at swimmer drop (DD), depth (DP), presence of calf (C) and whale’s behaviour at the time of the approach (B). One two-way interaction was also tested: vessel × distance of the boat from the whales at swimmer drop (V × DD). Repeated approaches targeting the same whale pods were excluded from the analysis to ensure independency of the samples. Therefore, only the first approaches by vessel or swimmers to whales were included in the analysis.

#### Diving time, respiration and reorientation rates

Average diving time (seconds), diving rate (dives × hour^-1^) and respiration rates (blows × individuals^-1^ × minute^-1^), and number of group reorientation events were compared between control and experimental (vessel and swimmer) samples. In addition, the proportion of time spent diving by the focal group was calculated (total dive time/encounter time) and compared between the three situations. When in presence of mother-calf pairs, the diving time of the mother was considered due to calves asynchronous diving behaviour. Data gathered from the research vessel in the absence of tourism activity (1,000 metres radius from the focal group) were considered as the control data set. Experimental data collected from the swim-with-whales vessel were divided in two groups; those where swimming with whales occurred (swimmers) and those where swimming with the whales did not occur (vessel). The analysis focussed on whale groups containing a calf that were approached in parallel by the primary swim-with-whales vessel (A). This focus was necessary due to the high number of potentially influential variables and the non-homogeneous sample. That is, whale mother-calf pairs were the preferred target by tour operators for swim-with-activities (68.8% of time spent with whales and 79.3% of swim time) and most of the encounters occurred when aboard swim-with-whales vessel A. Focal group follows were also filtered to include those with a minimum of 30 minutes of data, as per the control protocols. As a consequence, vessel and swimmer data for groups without a calf present were limited and the sample size was deemed too small to conduct valid analyses with enough statistical power.

The effect of water depth on response variables was also tested. The proportion of time spent diving was log-transformed as the models used for the analysis require a continuous dependent variable. Graphical validation tools were used to assess the underlying assumptions of variance homogeneity (plot residuals versus fitted values) and normality (quantile-quantile plot of the residuals) for average dive time, log-transformed proportion of time spent diving, diving and respiration rates. Shapiro-Wilk and Levene’s tests were also performed. No violations of normality were detected for control, vessel and swimmers’ data sets. Deviations from homoschedasticity were found for diving rate, average diving time and log-transformed proportion of time spent diving. Therefore, Weighted Least Squares (WLSQ) models were used to test if these response variables differed significantly between control, vessel and swimmers’ samples. ANOVAs and post-hoc Tukey’s tests were then conducted. A Linear Model (LM) was also used to investigate the hypothesis that respiration rate would not change between the three samples. Finally, the number of group reorientation events during the first 30 minutes of the encounter was modelled as a function of sample type using GLMs with negative binomial distribution and log link function. Statistical analyses were conducted using SPSS Statistic 24 software (IBM, Armonk, NY, US. 2016). For all analyses, statistical significance was assumed at α = 0.05 level.

## Results

Between July and October 2016 and 2017, 44 encounters with whales (28.3 hours) were recorded during 641 kilometres of survey effort across 20 days aboard research vessels. Nine encounters were excluded from the control data set as tour vessels interacted with the whales during the observation. Control observations lasted a total time of 19.1 hours (mean = 0.5, SD = 0.1 hours). During 36 days aboard swim-with-whale vessels (i.e., Vessel A and Vessel B), 2,516 kilometres were travelled and 146 separate encounters with whales (95.4 hours) were documented. During the two seasons of the study a total of 62 groups containing a calf were encountered (mother-calf pairs n = 46; mother-calf and escort = 16) versus 128 groups without a calf (single n = 62; duos n = 43; trios n = 11; four to nine individuals n = 12).

Vessel A and B spent an average of 2.6 hours per day in encounters with whales (4 encounters per day lasting 0.6 hours on average). Swimming activities were attempted 162 times with a total cumulative swim time of 24.8 hours. Vessel A conducted most of the swimming activities with whales (17.3 hours) over the two seasons. This tour operator focused particularly on mother-calf pairs (34.9 hours; 68.8% of the total encounter time) and most of the swim activities took place with whale groups containing a calf (13.8 hours; 79.3% of total swim time).

### Avoidance responses to vessel and swimmer approaches

**Fig 2:**
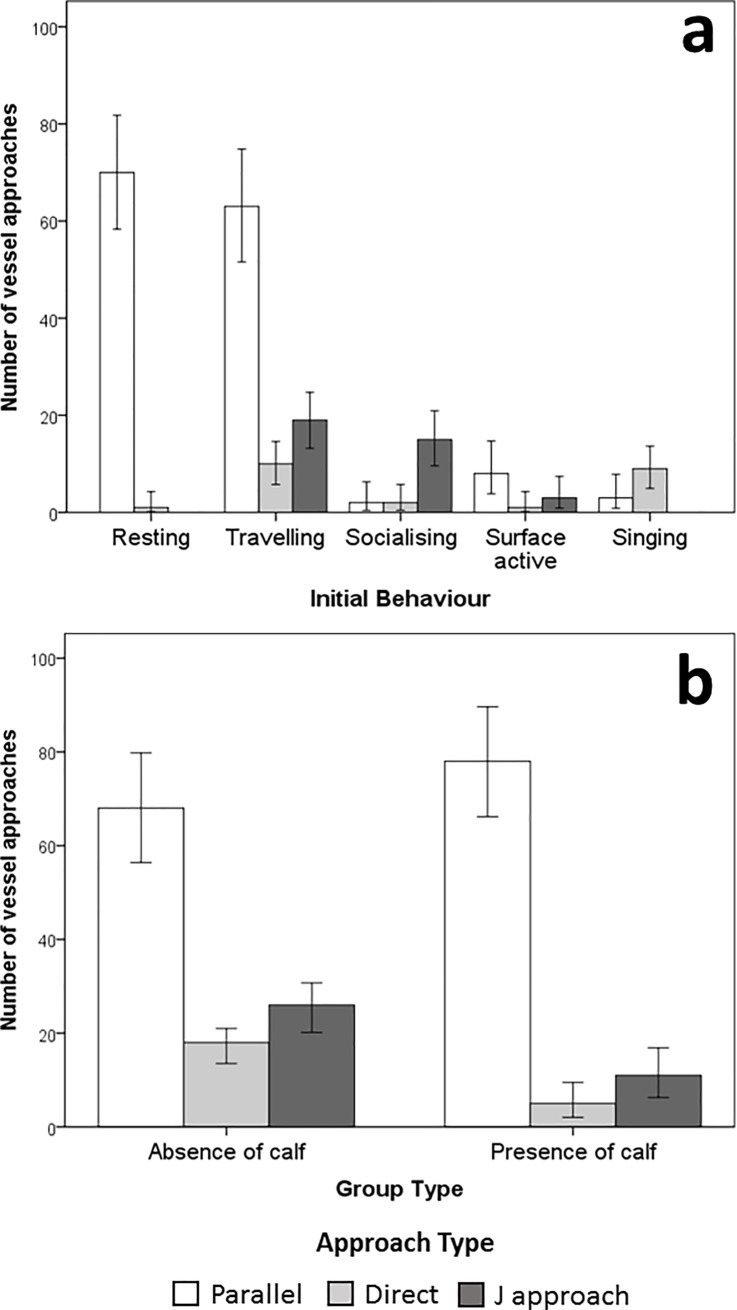
Number of documented swim-with-whale vessel approaches towards humpback whales (*Megaptera novaeangliae*) in Vava’u, Kingdom of Tonga. Shades indicate the approach type (parallel, direct, J). (**a**) On the horizontal axis is represented the initial behaviour of the targeted whales. Resting whales (n = 71) were approached almost exclusively in parallel (98.6%). Traveling whales (n = 92) were approached more frequently in parallel (68.5%) and with J approaches (20.7%). Socializing whales (n = 19) were approached more frequently with J approaches (89.5%). Direct approaches were used mainly with singing and travelling whales. (**b**) Horizontal axis indicates absence (n = 112) or presence of whale calves (n = 94) in the group targeted by the tour operators. Groups containing a calf were mainly approached in parallel (83.0%). J and direct approaches were used more frequently with groups without calves (39.3%). Error bars represent 95% confidence intervals.

Vessels A and B approached a focal group of whales a total of 206 times over the two seasons of data collection. The majority of approach types were parallel (70.9%, n = 146), while the J approach was used in 18.0% of the cases (n = 37). Direct approaches accounted for the remaining 11.1% (n = 23). Whales were recorded as actively avoiding vessel approaches for 33.5% of all approaches and the whales avoided the boat more frequently when the skipper used a J approach (67.6% of the J approaches elicited avoidance) in comparison to parallel (26.0% of the parallel approaches elicited avoidance) and direct approaches (26.1% of the direct approaches elicited avoidance). The choice of vessel approach type was influenced by the initial behavioural state and group composition of the whales (Fig 2A and 2B). Resting and travelling whales were approached most frequently in parallel (98.6% and 68.5% of the resting and travelling whales, respectively), while J approach types were more frequently conducted when whales were socializing (89.5%) and travelling (20.7%) (Fig 2A). Direct approaches were used mainly with singing whales (75.0% of the singing whales). However, singing individual whales were encountered on a few occasions (n = 6) and, therefore, were excluded from further statistical analysis due to the low sample size. Feeding behaviour was not observed during the study.

Vessels A and B approached whale groups containing a calf mostly using the parallel technique (83.0%; Fig 2B). Interestingly, in every instance recorded (n = 16), mother-calf pairs always responded by avoiding the vessel when direct and J approaches were used. The proportion of direct and J approaches increased significantly (Pearson’s *χ*^2^: *χ*^2^_2_ = 12.638, *P* = 0.002) for groups without a calf (Fig 2B).

The GLM that best fitted the data concerning the first approach on each group (n = 124) included the main effect approach type (AT) and the interaction vessel (V) × approach type (AT) (Table 4). The second model provided an equal amount of information but included also the main effect vessel (V) as predictor variable. That is, the model was less parsimonious and was discarded.

**Table 4:** Akaike’s Information Criterion (AIC) values and Variation AIC for best fitting models in comparison to full model.

Model	AIC	ΔAIC
**AT + (V × AT)**	132.919	0
**AT + V + (V × AT)**	132.919	0
**AT + V**	133.912	1.070
**AT + V + DI + DP + (V × AT) + (V × DI)**	137.494	4.575

**Fig 3:**
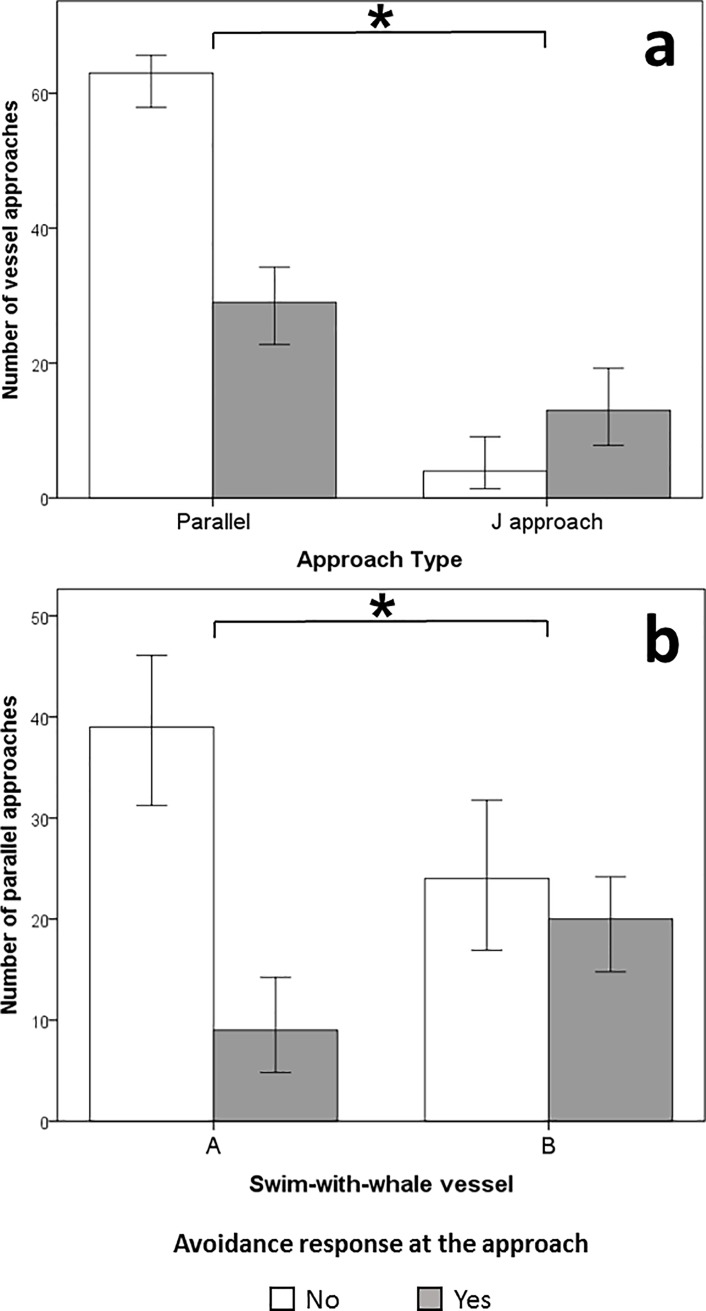
Number of documented swim-with-whale vessel first approaches towards groups of humpback whales (*Megaptera novaeangliae*) in Vava’u, Kingdom of Tonga. Shades represent the whale’s response (“avoidance”, “no avoidance”). (**a**) Comparison between whale responses to parallel (n = 92) and J (n = 17) approaches. 76.5% of J approaches elicited an avoidance response, while parallel approaches were significantly less avoided by whales (31.4%). (**b**) Whale responses to parallel approach by tour operator boat A (n = 48) and B (n = 44) are compared. Whale avoidance rate to vessel B parallel approach (45.4%) was significantly higher than the rate observed for vessel A (18.7%). Error bars represent 95% confidence intervals. Significant differences (P < 0.05) between avoidance rates detected by z-test for proportions are denoted by an (*).

In particular, J approaches corresponded to an avoidance rate (76.5%) significantly higher (95% CI: 20.7–69.2%, z = 3.50, *P* < 0.001) than for parallel approaches (31.4%; Fig 3A). Moreover, vessel B was avoided by whales more frequently than vessel A (95% CI: 7.8–45.6%, z = 2.75, *P* = 0.006) when using parallel approaches (Fig 3B).

The minimum boat distance (DI) reached during an approach (mean = 91.6 ± SE 7.9, SD = 82.1 metres) and depth (mean = 84.3 ± SE 10.5, SD = 109.6 metres) had no significant effect on vessel avoidance rates.

With regard to whale avoidance responses towards swimmers, a total of 162 swim approaches were observed. Whales showed avoidance behaviour to swimmers 35.5% of the time. GLM selection procedures did not highlight any significant effect of swimmer placement, vessel, vessel distance from the whales at swimmer drop, water depth, presence of calves or initial group behavioural state at the time of the first approach. That is, both models fitting data relative to “line abreast” and “in path” swimmer placement and data relative to “line abreast” alone were not significantly different from intercept only models (Likelihood Ratio for full models: *χ*^2^_6_ = 6.338, *P* = 0.386 and *χ*^2^_6_ = 3.371, *P* = 0.761, respectively).

### Diving time, respiration and reorientation rates

**Fig 4:**
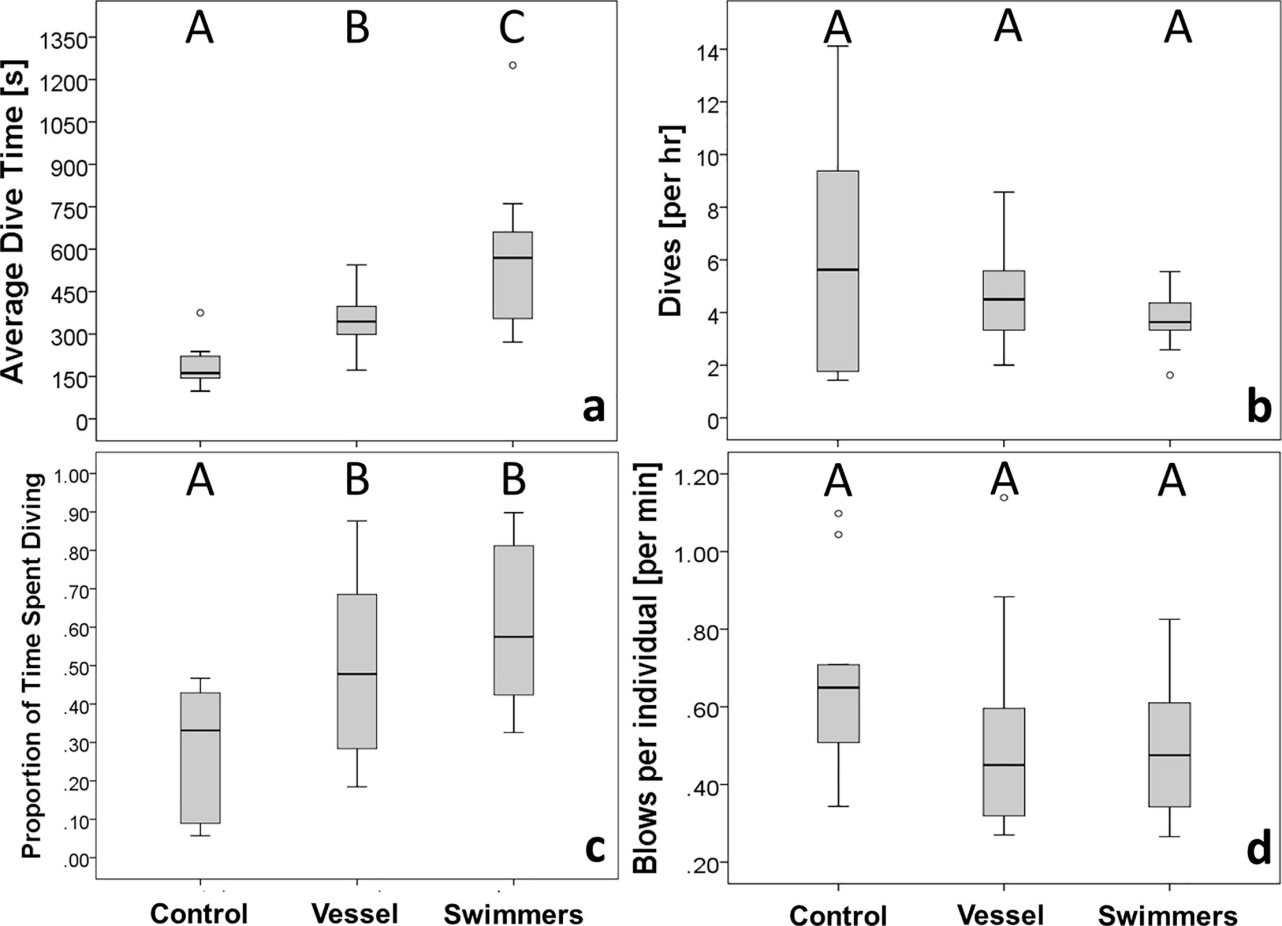
**Box plots representation of average dive time (a), diving rate (b), proportion of time spent diving (c) and respiration rate (d) in absence of tourism activity (control), in presence of swim-with-whale vessel A and during the swimming activities (swimmers) for humpback whales (*Megaptera novaeangliae*) mother-calf pairs in Vava’u, Kingdom of Tonga.** Capital letters indicate the results of Tukey’s post-hoc analysis. Different letters are associated with significant differences (*P* < 0.05).

The presence of vessel A and swimmers had a significant effect (*F*_*2*,*36*_ = 18.183, *P* < 0.001) on the dive time of a whale mother (i.e., A female adult whale with her calf present) (Fig 4A). Average dive time increased almost two-fold for tour vessel approach data sets (parallel approach type) (351 ± SE = 26 seconds) and three-fold during swimming activities (line abreast placement type) (561 ± SE = 73 seconds) when compared to control data sets (189 ± SE = 24 seconds). WLSQ model explained 52.4% of the variance. Although the diving rate decreased in the presence of a tour vessel (4.75 ± SE = 0.53 dives × hour^-1^) and in the presence of swimmers (3.77 ± SE = 0.28 dives × hour^-1^) with respect to controls (6.03 ± SE = 1.42 dives × hour^-1^), this change was not significant (*F*_*2*,*36*_ = 2.219, *P* = 0.125) (Fig 4B). However, whale mothers spent significantly more time diving (*F*_*2*,*36*_ = 5.462, *P* = 0.009) in the presence of both tour vessel and swimmer situations (vessel: 50.4 ± SE 6.7%; swimmers: 58.6 ± SE 6.0%) when compared with their time spent at the surface in a control situation (27.9 ± SE 5.2%) (Fig 4C). The model explained 24.9% of the variance in the data. Whale’s respiration rate (blows × individuals^-1^ × minute^-1^) decreased when whales were in the presence of vessel (0.51 ± SE = 0.07) and swimmers (0.49 ± SE = 0.04) compared to the control situation (0.67 ± SE = 0.07) (Fig 4D). However, this difference was not significant (*F*_*2*,*36*_ = 0.208, *P* = 0.140).

**Fig 5:**
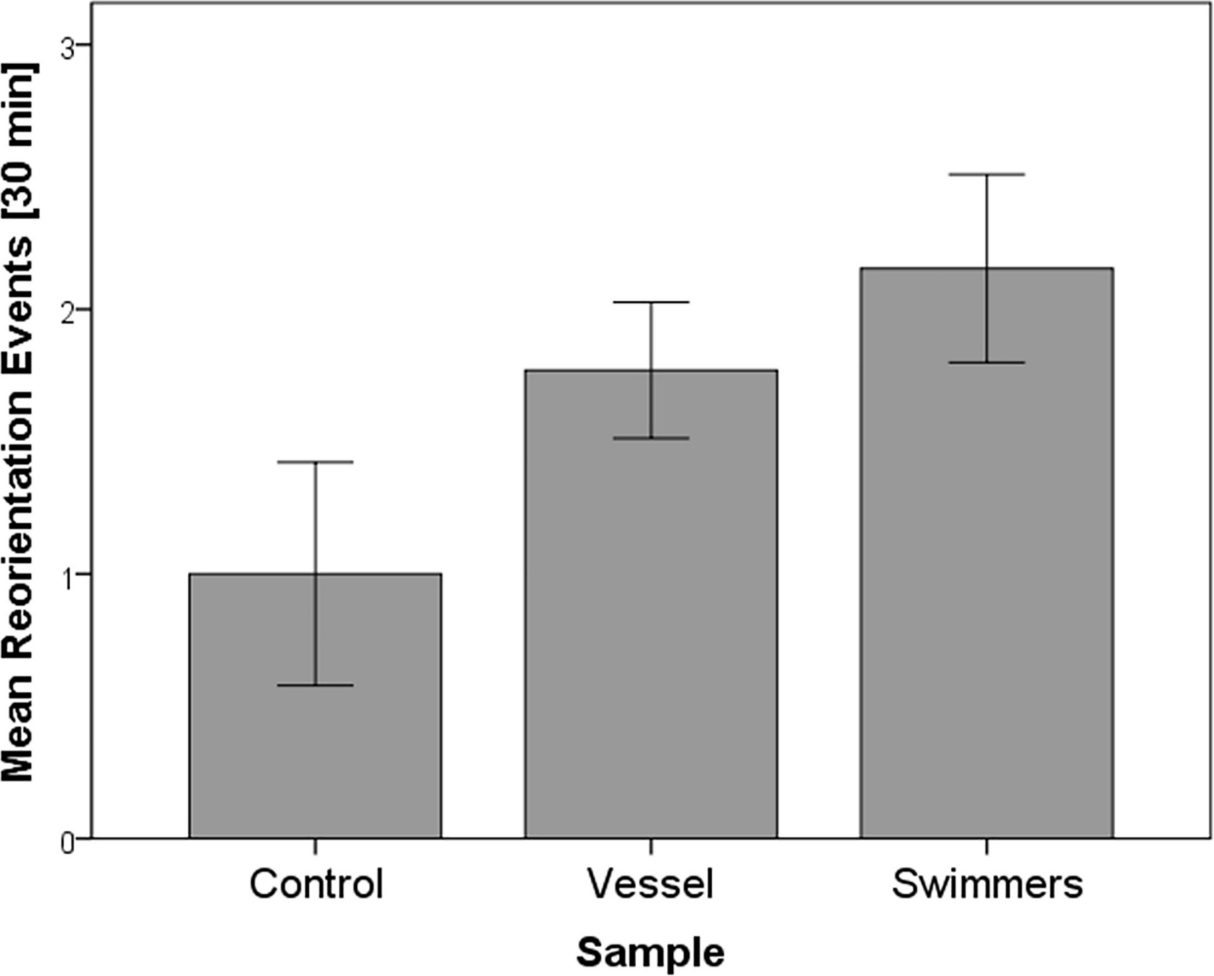
Mean values for re-orientation events observed in 30 minutes for mother-calf pairs in absence of tourism activity (control), in presence of a swim-with-whale vessel and swimmers (experimental) for humpback whales (*Megaptera novaeangliae*) mother-calf pairs in Vava’u, Kingdom of Tonga. Error bars represent the standard error of the mean.

Finally, the mean number of re-orientation events was higher when whale groups were approached by the tour vessel (1.77 ± SE 0.26) and swimmers (2.15 ± SE 0.35) than in their absence (1.0 ± SE 0.42; Fig 5). However, a GLM analysis did not detect any significant effect (Likelihood Ratio: *χ*^2^_2_ = 1.915, *P* = 0.384). Finally, no significant effect of water depth (mean = 66.4 ± SE 4.4, SD = 26.7 metres) was found for all the response variables investigated.

### Compliance with regulations

**Fig 6:**
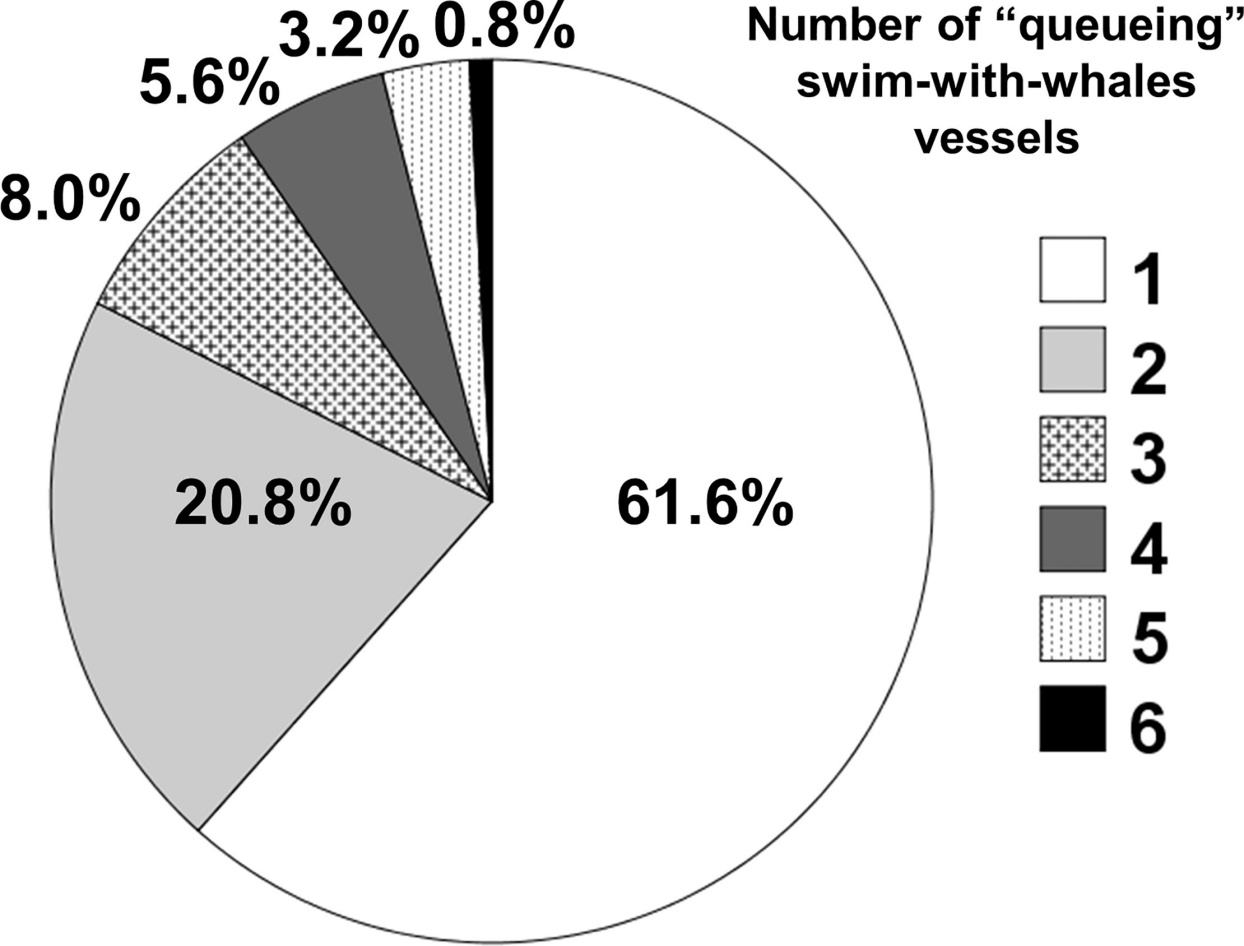
Percentage of encounters with humpback whales (*Megaptera novaeangliae*) in Vava’u, Kingdom of Tonga, during 2016 and 2017 seasons with number of tour vessels present in the area waiting to interact. The number includes the tour operator boat boarded by the researcher (vessel A or B).

Data collected from aboard tour vessels A and B indicated that 10.4% of encounters with whales (n = 146) lasted longer than 1.5 hours, the maximum interaction time permitted under the Tongan swim-with-whale regulations [54]. Furthermore, during 38.4% of the encounters recorded, additional tour vessels (up to six) queued within 300 metres of the whale group waiting to drop swimmers into the water with the same whales (Fig 6). This contravenes the Tongan swim-with-whale regulations, which requires a minimum whale resting time (no swimmers or vessels within 300 metres of the whales) of 1.5 hours between each interaction with tour vessels.

Vessels A and B were recorded approaching the focal group of whales closer than the minimum distance specified under the Tongan swim-with-whale regulations (10 metres for whale groups without a calf, 50 metres for whale groups containing a calf) in 13.1% of the cases documented (n = 206). In addition, during 19 of 162 swims observed (11.7%) more than five swimmers at the same time were dropped in the water (Tongan swim-with-whale regulations specify a maximum of four swimmers and one guide at one time). On one occasion, 10 swimmers were recorded in the water concurrently. Finally, on no occasions during this study were any official monitoring or enforcement vessels seen on the water observing whale tourism activities in Vava’u.

## Discussion

### Avoidance responses to vessel and swimmer approaches

The hypothesis that the tour vessel approach type does not affect humpback whale behaviour (specifically avoidance rates) was rejected by the data presented in this study. J approaches caused an avoidance response most frequently (+ 35.1%), while parallel approaches resulted in the least number of avoidance responses (Fig 3A). Cetaceans are known to respond with avoidance behaviour to erratic and fast movements of boats manoeuvring closely around them, especially in the middle of a group or directly in the path of their direction of travel. Not surprisingly, most whale-watching regulations and codes of conduct worldwide recommend the use of parallel approaches when interacting with whales and dolphins [31]. However, the Tongan regulations for commercial swimming activities do not explicitly provide indications on how to approach whales when dropping swimmers [54]. Despite this, the recommended parallel approach type was the most frequently used by Vava’u tour operators (70.9%).

This finding differs from what was reported by Sprogis et al. [18] during the first trials of swim-with-humpback whales in Ningaloo Marine Park, Western Australia. That is, at Ningaloo, tour operators interacted with humpback whales using mainly a J approach (89.8%) positioning swimmers in the path of travel of the whales. Several factors may explain this difference between the Tongan and Ningaloo whale swim operations. Western Australian swim-with-whale regulations do not permit swimming with groups containing calves [55]. In contrast, tour operators in Vava’u interact especially with mother-calf pairs (79.3% of total swim time). The predominant activity of a mother-calf pair in Vava’u is resting. This situation facilitates the use of a parallel approach by swim-with-whale tour operators. In addition, mother-calf pairs seem to be particularly sensitive to direct and J approaches and exhibit avoidance responses in all the cases (n = 16) observed in Vava’u. As a consequence, tour operators in Vava’u most likely use a parallel approach because it results in a higher probability of success for their swim-with customers. In contrast, and similar to Ningaloo, Vava’u swim-with-whale operators use a J approach more frequently when targeting whale groups without a calf (Fig 2B). This is likely to be related to the more common socialising and travelling behavioural states of whale groups without calves in Vava’u, and, potentially, also in Western Australia.

A further factor that may explain the difference between Vava’u and Ningaloo is the significance of their respective location in the whale’s annual migratory cycle. Ningaloo is situated in the Exmouth Gulf, which represents a resting ground for Western Australian humpback whales along their southward migration, especially for mother-calf pairs [56]. In contrast, the Vava’u archipelago is a breeding and calving ground for the Tongan humpback whale sub-population [39,41]. Consequently, Western Australian whales exhibit a different behavioural scenario with respect to their Tongan conspecifics, most probably because they are at a different stage of their annual migration.

In Vava’u, vessel approach type was not the only factor affecting whale avoidance rates. Whale focal groups displayed more frequent avoidance responses towards tour vessel B (+27.5%) when it approached using the parallel technique (Fig 3B), while J approaches caused a similar avoidance rate for both tour vessels. This finding may reflect differences between the design (vessel A = catamaran; vessel B = monohull) and/or propulsion type for each vessel (vessel A = gasoline fuelled outboard motors; vessel B = diesel fuelled inboard engines) or potentially the different hydrodynamic characteristics related to the hull shapes. An additional, potentially influential variable, may have been the longevity of vessel in the area as a swim-with-whales platform. Vessel A had been conducting swim activities in Vava’u for over a decade, while vessel B was only in the second year of operations. Research in other areas has shown that whales can become habituated [57] towards a vessel that has been operating in close proximity for several years (e.g., [58,59,60]).

Factors such as the tour vessel type, swimmer placement and vessel distance at swimmer drop had no significant effect on the avoidance rate. These findings contrast with research conducted by Constantine (2001) on bottlenose dolphins (*Tursiops truncatus*) in the Bay of Islands, New Zealand, who found higher levels of avoidance towards “in path” swimmer placements when compared with “line abreast” and “around the boat” placements. Any assessment of swimmer placement technique is confounded by the presence and manoeuvring of the vessel that is being used for placing the swimmers in the water [8]. While the findings of our study did not find any significant relationship between swimmer placement technique and whale avoidance behaviour, other variables which may be influential were not tested. For example, the behaviour of the swimmers in the water and their distance to the whales might influence whale responses. Unfortunately, such variable could not be assessed accurately from the tour vessel as an observation platform. Interestingly, a study into the effects of swimmer behaviour on humpback whales in the Ha’apai island group, Kingdom of Tonga, indicated that whales moved away from swimmers significantly earlier when swimmers were splashing instead of being calm during their in-water encounters with the whales [8].

### Diving time, respiration and re-orientation rates

The hypotheses that swim-with-whales tourism activities had no effect on humpback whale dive time as well as on the proportion of time spent diving was rejected for mother-calf pairs. Results indicated that whale mothers increased their average dive duration two-fold when in the presence of tour vessel and three-fold when in the presence of swimmers in comparison to control observations (Fig 4A). In addition, the proportion of total time spent diving during tourism encounters doubled in the presence of swimmers and tour vessel (Fig 4C). That is, our study provided evidence that whale mothers in Vava’u adopt a vertical avoidance strategy in response to swim-with-whales vessel approaches and, in particular, to swimmer approaches. Similar findings have been reported for humpback whales exposed to whale-watching vessels in other breeding grounds [20,24] and also in humpback whale migratory corridors [23]. Although the biological consequences of such avoidance behaviour is not clear, such strategies may increase energy expenditure for the lactating mother and for her dependent offspring [61,62]. Evidence from other parts of the world suggests it is likely that such behaviour is detrimental for the affected whales. For instance, Braithwaite et al. [63] estimated that the increase of swimming speed and the reduction in the time spent resting for mother-calf pairs can result in a significant decrease in the calf’s growth rate.

Humpback whales in Western Australia, on the other hand, reacted to swimming activities by decreasing their average dive duration and increasing the deviation index (the mean of turning angles between consecutive positions during the follow) with respect to the approaching vessel [18]. Although our study documented an increase of reorientation events in presence of a tour vessel and swimmers (Fig 5), this finding was not statistically significant. However, reorientation may not be the only observable behavioural change that might indicate a reaction to the presence of vessels and/or swimmers. Whale swim speed, for instance, could also be investigated as potential indicator of horizontal avoidance. Scheidat et al. [26], for example, documented that humpback whales almost doubled their swim speed in the presence of whale-watching boats while the number of reorientation events did not increase significantly. An additional study on humpback whales in their breeding grounds off Bahía Málaga, Colombia, found an increase in both swim speed and reorientation rates, and a decrease of whale respiration rates in response to the presence of vessels [27]. During our research in Vava’u, a slight decrease in whale respiration rates in the presence of tour vessels and swimmers was recorded, but this was not statistically significant (Fig 4D). It is important to note that the absence of a statistically significant change at the group level may not be synonymous with the lack of changes at the individual level, especially if different age classes are considered [19]. In our study, the group respiration rate was calculated by dividing the total number of respiration events by the total number of whales in the group. As a consequence, while this may be representative for whales’ breathing synchronously, changes in respiration rates for individuals, which have different respiration patterns from the wider group, as is the case with calves, can be underestimated.

Unfortunately, it was not possible to investigate the effects of swimming on focal groups of whales not containing calves because it occurred so rarely that the sample size was too small for any valid statistical analysis. This low sample size was also a consequence of swim-with-whale tour operators’ preference for targeting mother-calf pairs in Vava’u.

### Compliance with regulations

We documented low levels of compliance with Tongan key swim-with-whale regulations in Vava’u [54] and our findings support those reported by Walker and Moscardo [9]. In particular, during 38.4% of the encounters tour operators did not comply with the minimum rest time between interactions with tour vessels, which is stipulated as 1.5 hours under the 2013 regulations. Up to six vessels were observed waiting (“queueing”) to interact with a focal whale group, which already had a tour vessel interacting with it. These queueing vessels would then move in immediately after the original vessel departed the area or, in some cases, they would alternate with the original vessel by placing swimmers in the water as soon as the initial vessel swimmers exited the water. In addition, on many occasions (38.4% of the encounters), tour vessels were queued up within 300 metres from the whales waiting to move in to commence in-water interactions with whales as soon as the tour vessel boarded by the researchers departed the area. Up to 28 commercial swim-with-whales vessels were counted on the water simultaneously during the peak of the 2017 season. That is, the level of exposure of humpback whales to swim-with tourism activities in Vava’u is extremely high during daylight hours, both in terms of the number of tour boats and the time spent with the whales (mean = 2.6 hours per day per vessel).

While compliance with minimum rest-times was poor, adherence with other regulations was higher. For example, the maximum number of five swimmers (including the guide) in the water at a single time was respected by tour operator vessels A and B in 88.3% of the interactions. Notably, breaches to this regulation generally occurred when five swimmers were on board the tour boat. Tour operators A and B opted to let all the swimmers enter the water with the guide (thereby exceeding the five person maximum) instead of dividing them in two groups and reducing the interaction time for each group. On one occasion, two groups of five swimmers were in the water at the same time as a consequence of the presence of a second tour operator initiating the swimming activity before the first operator could retrieve its participants. Despite this, the four-to-one swimmers-to-guide ratio seemed to be adhered to most of the time by tour operators. Therefore, compliance with this regulation was generally high, as documented also by Sprogis et al. [18] in Western Australia.

The minimum distance a vessel should approach the whales under the regulations in Tonga (10 metres for whale groups without a calf, 50 metres for whale groups containing a calf) was adhered to by vessels A and B in 86.9% of their approaches. This level of compliance in Vava’u was higher than that reported for Ningaloo, Western Australia (68.5%) [18]. However, Western Australia regulations [55] are more restrictive than in Tongan waters (i.e. 50 metres for parallel approach, 150 metres for J approach). It is possible that adhering to closer distances as in the Tongan regulations might be easier for operators [64], especially considering that boat crews rarely use laser rangefinders to assess the distance from the whales during their approaches [18]. Finally, it is important to note that the influence of researchers on board the tour vessels cannot be excluded, which may have resulted in operators being more likely to comply with license conditions. Moreover, data were collected only for two operators in Vava’u that accepted to host researchers onboard. Therefore, no information is available to document how the other 18 operators behaved during the encounters with whales in terms of approach distance, duration of the encounters and number of swimmers simultaneously in water.

## Conclusions

This study highlights that both observing and swimming activities cause avoidance responses from humpback whales in Vava’u, Kingdom of Tonga. In particular, mother-calf pairs showed significant vertical avoidance responses, with humpback whale mothers diving for significantly longer periods of time in the presence of vessels and swimmers. Whether the short-term behavioural responses observed in Vava’u humpback whales could cause a long-term detrimental effect at the population level is unknown and needs further investigation. Again, evidence from other studies on the effects of cetacean based tourism suggests that the findings from our study in Vava’u should be cause for concern.

This study also documented low levels of compliance to Tongan regulation with regard to the minimum resting time for whales between interactions with commercial vessels.

The Tongan sub-population of humpback whales still shows little evidence of recovery after the cessation of whaling, in contrast to other regions such as the East and West coasts of Australia [40–42]. Moreover, the Vava’u island group represents one of the most important breeding and calving grounds for this population. That is, humpback whales give birth and raise calves in the sheltered waters of the archipelago, readying them for the long southward migration to Antarctica [39]. The behavioural responses documented in this study underlie the risk of detrimental effects on this population of whales targeted by swim-with-whale tourism. The rapid growth of swim-with-whales industry experienced by Vava’u over a short period of time [5] and the tour operator focus on mother-calf pairs is concerning, especially in the light of the poor compliance with regulations and the lack of enforcement of formal regulations documented by this study (Tongan Ministry of Tourism, personal communication, October 8, 2017). Some management actions have been recommended by the authors and are currently under consideration by the Tongan Ministry of Toursim:

- Focus on increasing compliance with the existing whale-watching regulations;
- Reduce the number of swim-with-whales licensed vessels;
- Introduce a break time in the middle of the day (eg., from12 to 2 pm) when swim-with-whales operations are not allowed.

Overall, our findings reinforce the urge for a more cautious and effective approach to the management of swimming activities with humpback whales, both for Tongan authorities and other governments willing to permit these activities.
